# Endoscopic ultrasound-guided drainage for a delayed splenic abscess caused by electrocoagulation syndrome after endoscopic resection of small gastric submucosal tumor

**DOI:** 10.1055/a-2780-6124

**Published:** 2026-02-09

**Authors:** Liliangzi Guo, Jun Yao, Juan Cao, Su Luo, Ruiyue Shi, Lisheng Wang, Zhenglei Xu

**Affiliations:** 1 12387 Department of Gastroenterology, Shenzhen Peopleʼs Hospital, Shenzhen, China

**Fig. 1:**
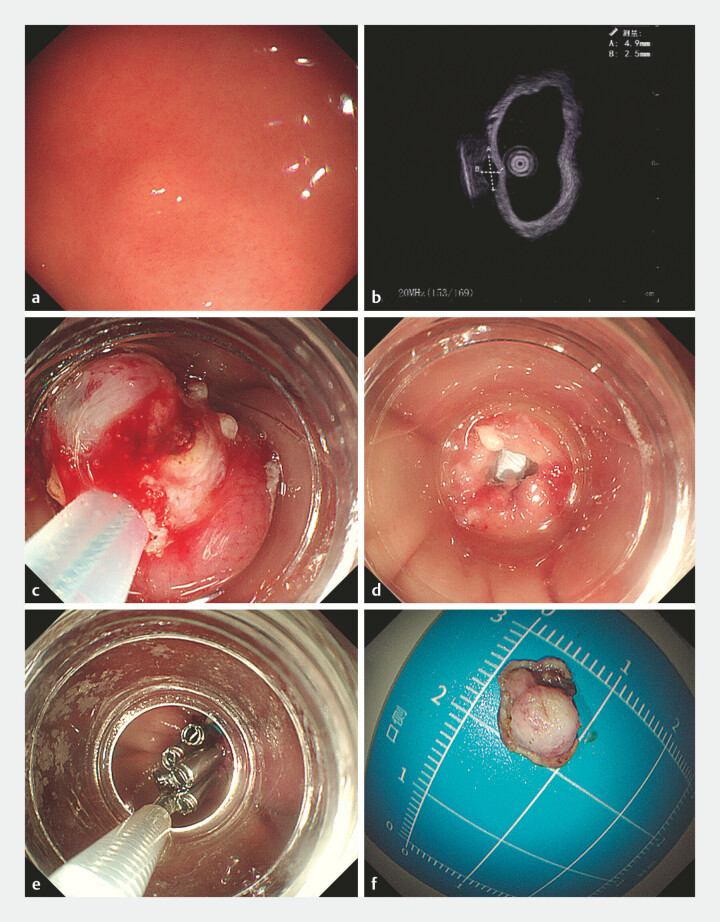
Procedure of the endoscopic resection. **a** A gastrointestinal stromal tumor in gastric fundus; **b** EUS showed that the tumor originated from the muscularis propria, with a length of 5 mm; **c** endoscopic resection using a cap-assisted method; **d** no perforation and bleeding occurred; **e** the wound was closed by clips and a ligating device; **f** the tumor was completely resected. EUS, endoscopic ultrasonography.

**Fig. 2:**
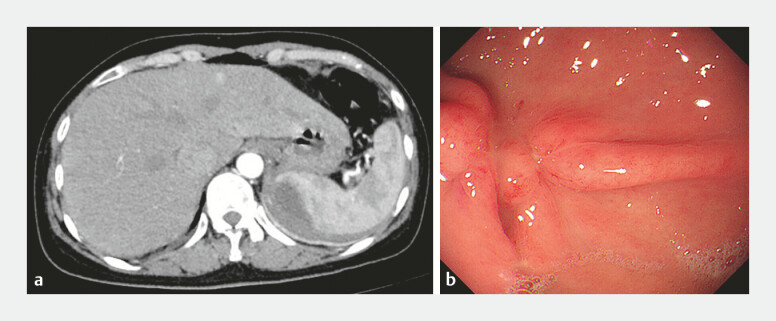
Two weeks after the operation, the patient was admitted with abdominal pain and high fever. **a** An abdominal CT scan revealed a splenic abscess; **b** second-look endoscopy found no delayed perforation or bleeding. CT, computed tomography.

**Fig. 3:**
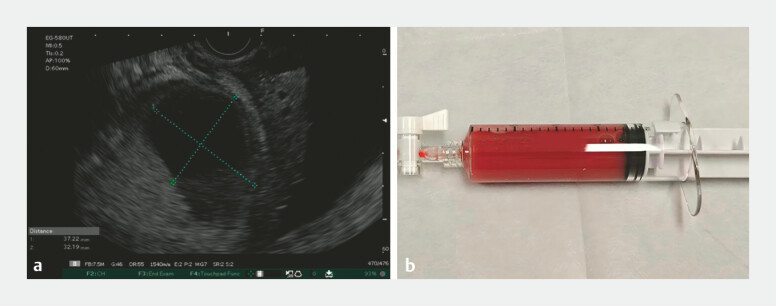
**a** EUS revealed an anechoic lesion at the spleen; **b** 20 mL of the bloody fluid was extracted under EUS guidance. EUS, endoscopic ultrasonography.

A 51-year-old woman underwent an endoscopic resection of a gastrointestinal stromal tumor in our endoscopy center. Endoscopic ultrasonography (EUS) showed that the tumor originated from the muscularis propria in gastric fundus, with a length of 5 mm. Resection was performed by using a cap-assisted method. Perforation did not occur during the operation, and then, the wound was closed by clips and a ligating device ( Fig. 1 ). Unexpectedly, the patient was admitted with abdominal pain and high fever 2 weeks after the operation. Laboratory tests found that the white blood cell count was 8.95 × 10 ^9^ /L, the neutrophil count was 88.9%, the hemoglobin level was 112 g/L, the C-reactive protein level was 77.5 mg/L and the procalcitonin level was 4.91 ng/ml. An abdominal computed tomographic (CT) scan revealed a splenic abscess ( Fig. 2 **a** ). Second-look endoscopy was performed, and no delayed perforation or bleeding was observed ( Fig. 2 **b** ). There was no accessible path for CT-guided drainage, so we performed EUS-guided drainage to treat the sepsis ( Video 1 ). EUS revealed an anechoic lesion of approximately 3.7 cm in size at the spleen ( Fig. 3 **a** ). Under EUS guidance, 20 mL of the bloody fluid was extracted for relief and further examination ( Fig. 3 **b** ). The microbial culture confirmed infection with *Stenotrophomonas maltophilia* . Abdominal pain and fever were significantly resolved on the day after the procedure. Repeated abdominal CT showed a significant resolution of the splenic abscess.

EUS-guided drainage for a splenic abscess after endoscopic resection of the small gastric submucosal tumor. EUS, endoscopic ultrasonography.Video 1

Development of the splenic abscess in the absence of leakage is extremely rare. We consider that thermal transmural injury caused by electrocoagulation syndrome may be the reason. To our knowledge, this is the first report of delayed splenic abscess after endoscopic resection of small gastric submucosal tumor. EUS-guided drainage has emerged as a viable therapeutic modality for splenic abscess 1 2 . When combined with antibiotic therapy, the patient eventually recovered and avoid unnecessary splenectomy. This case sends a reminder of the unusual complication of endoscopic intervention and provides a minimally invasive and alternative option for treating splenic abscess.

Endoscopy_UCTN_Code_CPL_1AH_2AZ
